# Coronary Artery Bypass Grafting in Patients with Acute Myocardial Infarction and Cardiogenic Shock

**DOI:** 10.31083/j.rcm2307237

**Published:** 2022-06-24

**Authors:** Christina Grothusen, Christine Friedrich, Ulysses Ulbricht, Jette Meinert, Tim Attmann, Katharina Huenges, Christoph Borzikowsky, Assad Haneya, Jan Schoettler, Jochen Cremer

**Affiliations:** ^1^Department of Cardiovascular Surgery, University Hospital Schleswig-Holstein, 24105 Kiel, Germany; ^2^Medizinische Klinik I, St. Johannes Hospital Dortmund, 44137 Dortmund, Germany; ^3^Institute of Medical Informatics and Statistics, Kiel University, University Hospital Schleswig-Holstein, 24105 Kiel, Germany

**Keywords:** cardiogenic shock, acute myocardial infarction, CABG

## Abstract

**Objective:**

Acute myocardial infarction (AMI) complicated by cardiogenic shock (CS) remains associated with a high rate of mortality and disabling morbidity. Coronary artery bypass grafting (CABG) is seldom considered in this setting due to the fear of peri-operative complications. Here, we analysed the outcome of CS patients undergoing CABG within 48 hours after diagnosed with AMI.

**Methods:**

A single-center, retrospective data analysis was performed in 220 AMI patients with CS that underwent CABG within 48 hours between 01/2001 and 01/2018.

**Results:**

141 patients were diagnosed with ST-elevation myocardial infarction (STEMI), 79 with non-STEMI (NSTEMI). Median age was 67 (60; 72) for STEMI, and 68 (60.8; 75.0) years for NSTEMI patients (*p* = 0.190). 52.5% of STEMI patients and 39.2% of NSTEMI patients had suffered from cardiac arrest (CA) pre-operatively (*p* = 0.049). Coronary 3-vessel disease was present in most patients (78.0% STEMI vs 83.5% NSTEMI; *p* = 0.381). Percutaneous coronary interventions (PCI) were performed in 32.6% STEMI and 27.8% NSTEMI patients (*p* = 0.543) prior to surgery. Time from diagnosis to surgery was shorter in STEMI patients (3.92 (2.67; 5.98) vs 7.50 (4.78; 16.74) hours; *p *< 0.001). A complete revascularization was achieved in 82.3% of STEMI and 73.4% of NSTEMI cases (*p* = 0.116). Post-operative low cardiac output occurred in 14.2% of STEMI vs 8.9% of NSTEMI patients (*p* = 0.289). The rate of cerebrovascular injury–including hypoxic brain damage was 12.1% for STEMI and 10.1% among NSTEMI patients. (*p* = 0.825). 30-day mortality was 32.6% after STEMI vs 31.6% in NSTEMI cases (*p* = 0.285).

**Conclusions:**

In contrast to the discouraging data concerning the role of PCI in AMI patients with CS and complex coronary artery disease, CABG may represent a treatment option worth considering.

## 1. Introduction

Percutaneous coronary interventions (PCI) are nowadays performed with a high-rate of initial peri-procedural success in the majority of patients with acute myocardial infarction (AMI) complicated by cardiogenic shock (CS). Nevertheless, recent data demonstrated that AMI patients with CS still suffer from a devastatingly limited prognosis due to persisting cardiac low-output syndrome and severe neurological injury. In fact, mortality rates of these patients do not seem to have changed during the last decades despite the advances in interventional cardiology and the routine use of extracorporeal life support systems (ECLS) or Impella® [1, 2, 3]. According to current guideline recommendations, CABG should be performed without delay in CS cases that cannot be reasonably treated by PCI [4, 5, 6]. However, only a minority of patients in clinical practice undergoes immediate or staged operative revascularization, eventually. The aversion towards CABG in this setting is shared by cardiologists and heart surgeons alike. It is based on the belief that time to surgery would be too long to save ischemic myocardium while the risk of peri-operative complications would most likely jeopardize any chance left of a satisfactory quality of life or life itself. As a consequence, the study of White *et al*. [7] and colleagues, that was published more than ten years ago with data obtained during the 1990s, has remained the last randomized, prospective evidence, that operative myocardial revascularization of patients with CS is associated with a beneficial outcome. As the initiation of prospective studies would need the unlikely support of both, cardiologists and heart surgeons, retrospective data analysis currently remains the only possibility to elucidate the role of CABG in these particular patients. There are only a few, contemporary surgical studies available regarding this topic. However, these works indicate that CABG is a valid therapeutic option with surprisingly good results, although surgical patients after failed PCI are considered to be the most vulnerable [8, 9]. In order to improve and extend the public information available on this topic, we analyzed data obtained from our institutional registry that currently includes over 1300 patients that underwent CABG within 48 hours after diagnosed with AMI [10]. The study presented here focuses on the fate of patients with CS.

## 2. Material and Methods

### 2.1 Data Source

**Fig. 1.**
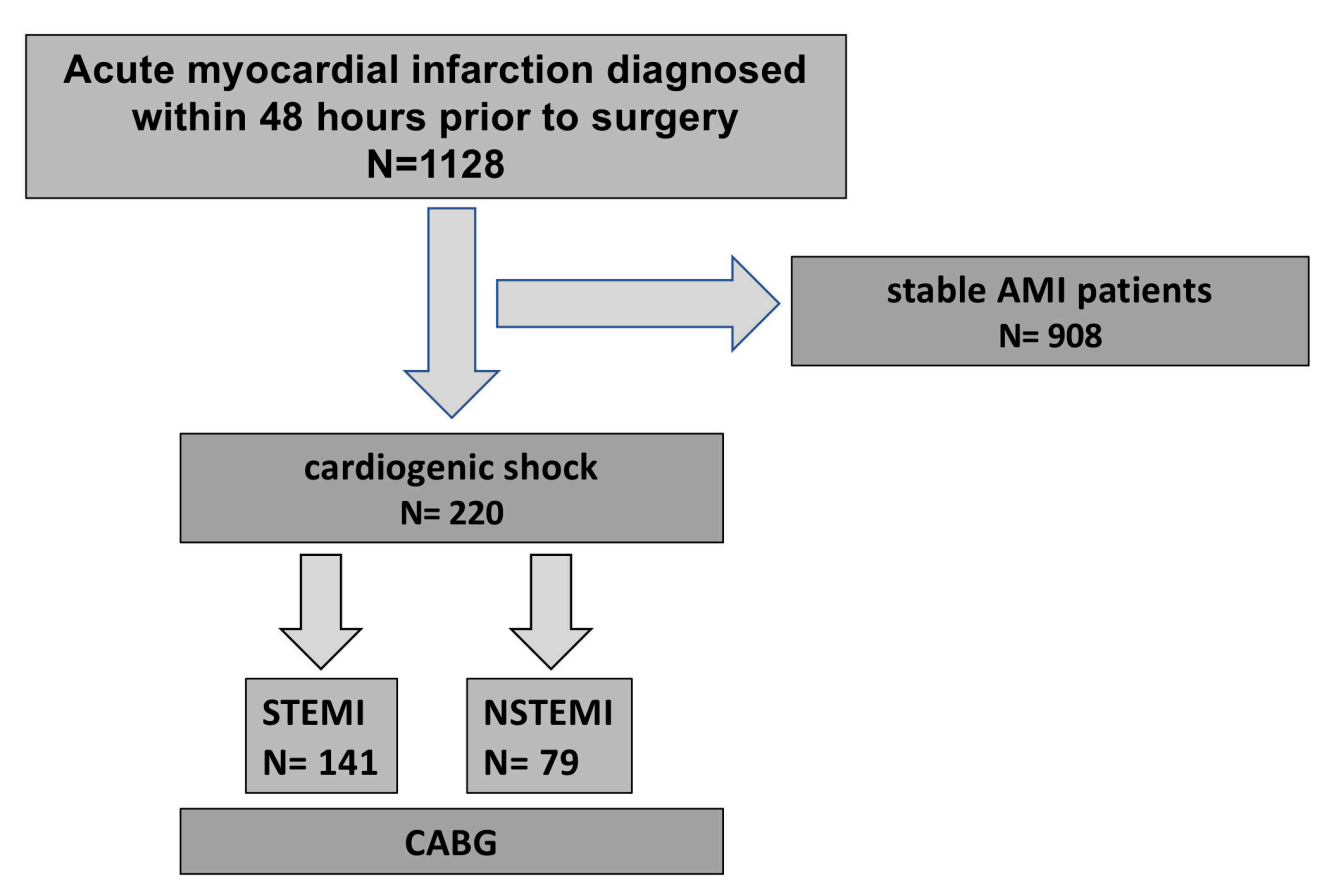
**Study design**. AMI, acute myocardial infarction; STEMI, ST-elevation myocardial infarction; NSTEMI, non-STEMI.

Between January 2001 and January 2018, 1128 consecutive, unselected patients with AMI underwent CABG at our institution within 48 hours after the diagnosis of AMI had been made. Of those, 220 patients were in cardiogenic shock (CS) pre-operatively (Fig. 1). A patient was considered to be in CS if persistent hypotension (<90 mmHg systolic blood pressure for at least 30 minutes) had been documented and/or a continuous infusion of catecholamines had been administered to maintain a systolic blood pressure >90 mmHg prior to surgery. In addition, information about clinical signs of pulmonary congestion, and/or signs of impaired organ perfusion with altered mental status, cold and clammy skin and limbs, oliguria with a urine output of less than 30 mL/h, or an arterial lactate level of more than 2.0 mmol/L had to be available in order to define a patient as being in CS pre-operatively. The general discrimination between ST-elevation myocardial infarction (STEMI) and non-STEMI (NSTEMI) if not clearly stated by the referring cardiologists was made following current guideline recommendations [4, 5]. The time point of STEMI or NSTEMI diagnosis was used for the determination of the time interval between diagnosis and CABG. Patients in need of combination procedures were excluded.

### 2.2 Surgical Management

A standard median sternotomy and cardiopulmonary bypass (CPB) was used in all but one patient. Myocardial arrest was obtained with cold blood cardioplegic solution applied antegrade via the ascending aorta. If insufficient myocardial protection was anticipated by this approach, antegrade cardioplegia was combined with retrograde application. The choice of graft was left at the discretion of the surgeon in charge. If bleeding was not a concern, acetylsalicylic acid was administered orally starting on post-operative day one. CABG was performed under dual platelet therapy regardless of the P2Y12 inhibitor used.

### 2.3 Statistical Analysis

Nominal and ordinal data were described as absolute and relative frequencies and compared using χ^2^-test or Fisher’s exact test, if one of the expected values in the 2 × 2 table was less than 5. The interval and ratio data were tested for normal distribution by Kolmogorov-Smirnov test. Normally distributed demographic and clinical patient data are presented as mean and standard deviation and compared using unpaired *t*-test. Not normally distributed data were described as median and 25th and 75th percentiles and compared using Mann-Whitney U-test. Parameters with a significant relation to 30-day mortality in the univariate analyses were included into multiple logistic regression analysis to assess their relative impact (adjusted odds ratio), except for EuroScore II due to its collinearity with several other predictor variables. The survival curves were estimated from right-censored data by Kaplan-Meier analyses. The survival of patients with STEMI and NSTEMI was compared by log-rank test. All statistical tests were performed two-tailed at a significance level of 5%. Statistical analysis was conducted using Statistical Package for Social Sciences (SPSS, Version 15.0, SPSS Inc., Chicago, IL, USA) and R (Version 3.3.2, Microsoft, Redmond, WA, USA).

## 3. Results

### 3.1 Baseline Characteristics

Of the 220 CS patients that underwent CABG within 48 hours after diagnosed with AMI, significantly more patients had been diagnosed with STEMI than NSTEMI (141 (64.1%) vs 79 (35.9%); *p* = 0.0001). As shown in Table 1, we did not observe significant differences in age (67 (60; 72) years in the STEMI vs 68 (60.8; 75.0) years in the NSTEMI group (*p* = 0.190) or proportion of female patients (27 (19.1%) female STEMI vs 21 (26.7%) female NSTEMI patients; *p* = 0.234). The distribution of cardiovascular risk factors was similar between the groups as was the calculated EuroScore (ES) II score (STEMI ES II 15.58 (11.80; 22.37) vs NSTEMI ES II 16.66 (11.76; 27.46), *p* = 0.465). More STEMI patients had undergone systemic thrombolysis (24 (17.0%) vs 4 (5.1%), *p* = 0.006) as well as intra-aortic balloon pump (IABP) implantation prior to surgery (75 (53.2%) vs 27 (34.2%), *p* = 0.08). Most patients were ventilated upon arrival in the operating theatre (87 (61.7%) STEMI vs 47 (59.5%) NSTEMI patients; *p* = 0.775). Slightly more STEMI patients had suffered from cardiac arrest (CA) pre-operatively (74 (52.5%) vs 31 (39.2%); *p* = 0.049). Time to surgery was significantly shorter in STEMI patients (3.92 (2.67; 5.98) hours vs 7.50 (4.78;16.74) hours, *p *< 0.001).

**Table 1.** **Baseline characteristics**.

Parameter	STEMI (n = 141)	NSTEMI (n = 79)	*p*-value
Age (years)	67 (60; 72)	68 (60.8; 75.0)	0.190
Sex (female)	27 (19.1)	21 (26.7)	0.234
BMI	26.12 (24.64; 29.39)	26.6 (24.2; 3.2)	0.737
IDDM	12 (8.5)	10 (12.8)	0.351
Arterial hypertension	98 (69.5)	57 (72.2)	0.578
hyperlipidemia	56 (39.7)	29 (36.7)	0.758
smoking	50 (35.5)	29 (36.7)	0.884
Renal impairment	24 (17.6)	20 (25.3)	0.161
Renal replacement therapy	2 (1.4)	3 (3.8)	0.353
LV-function <30%	39 (29.8)	23 (29.1)	0.876
EuroScore II (%)	15.58 (11.80; 22.37)	16.66 (11.76; 27.46)	0.465
Pre-hospital thrombolysis	24 (17.0)	4 (5.1)	0.006
IABP pre-operatively	75 (53.2)	27 (34.2)	0.008
Intubated on admission	87 (61.7)	47 (59.5)	0.775
inotropic support	120 (85.1)	61 (77.2)	0.146
Cardiac arrest	74 (52.5)	31 (39.2)	0.049
Lactate levels ≥2 mmol/L	64 (57.1)	33 (41.8)	0.671
Time to surgery (h)	3.92 (2.67; 5.98)	7.50 (4.78; 16.74)	<0.001
Prior stroke	10 (7.1)	3 (3.8)	0.386
Prior myocardial infarction	23 (16.4)	9 (11.4)	0.425
Prior cardiac surgery	1 (0.7)	1 (1.3)	1.000

BMI, Body mass index; IDDM, Insulin-dependent diabetes mellitus; LV, left ventricle; IABP, intra-aortic balloon pump. Values are given as n (%), mean ± SD or median (range).

### 3.2 Preoperative Details

110 (78.0%) STEMI patients and 66 (83.5%) of NSTEMI patients suffered from coronary 3-vessel disease (*p* = 0.381). Left main disease (≥50% stenosis) was present in 71 (50.4%) STEMI cases and 38 (48.1%) NSTEMI cases (*p* = 0.779). 46 (32.6%) STEMI patients and 22 (27.8%) NSTEMI patients had undergone a percutaneous treatment attempt within the 48 hours time frame prior to CABG (*p* = 0.543). Among these patients, PCI had been unsuccessful in 16 (34.8%) STEMI and 11 (50.0%) NSTEMI patients (*p* = 0.292) and/or was associated with a complication in 24 (52.2%) STEMI compared to 9 (40.9%) of NSTEMI cases (*p* = 0.444) (see Table 2).

**Table 2.** **Preoperative details**.

Parameter		STEMI (n = 141)	NSTEMI (n = 79)	*p*-value
1-VD		9 (6.4)	3 (3.8)	0.544
2-VD		22 (15.6)	9 (11.4)	0.427
3-VD		110 (78.0)	66 (83.5)	0.381
LM stenosis		71 (50.4)	38 (48.1)	0.779
LM thrombosis		12 (8.5)	3 (3.6)	0.266
LM dissection		9 (6.4)	4 (5.1)	0.775
PCI		46 (32.6)	22 (27.8)	0.543
	successful but incomplete revascularization	7 (15.2)	3 (13.6)	1.000
	failure	16 (34.8)	11 (50.0)	0.292
	complication	24 (52.2)	9 (40.9)	0.444
Stent implantation		17 (12.1)	5 (6.3)	0.242
	DES	7 (41.2)	1 (20.0)	0.613
	BMS	7 (41.2)	0 (0.0)	0.314
	unknown	3 (17.6)	4 (80.0)	0.020
Acetylsalicylic acid + clopidogrel		27 (19.1)	18 (22.8)	0.601
Ticagrelor		2 (1.4)	4 (5.1)	0.191
GP IIB/IIIa Antagonist		47 (33.3)	21 (26.6)	0.361

VD, vessel disease; LM, left main; PCI, percutaneous coronary intervention; DES, drug-eluting stent; BMS, bare-metal stent; GP, Glycoprotein. Values are expressed as n (%).

### 3.3 Intraoperative Details

We did not observe differences in total procedure time (STEMI: 216.2 ± 56.2 minutes vs NSTEMI: 225.9 ± 49.8 minutes, *p* = 0.205), but cross-clamp time was significantly shorter in STEMI patients (54 (42.5; 69.5) minutes vs 59.5 (49.8; 71.3) minutes; *p* = 0.028). The median number of distal anastomoses was similar in STEMI (3 (3; 4)) compared to NSTEMI (3 (3; 4)), (*p* = 1.000). The left internal thoracic artery was the arterial graft most often used in 82 (58.2%) STEMI and 53 NSTEMI cases (67.1%), (*p* = 0.313). The rate of complete revascularizations was 82.3% in STEMI and 73.4% in NSTEMI patients (*p* = 0.116) (see Table 3).

**Table 3.** **Intraoperative details**.

Parameter	STEMI (n = 141)	NSTEMI (n = 79)	*p*-value
Operation duration (min)	216.2 ± 56.2	225.9 ± 49.8	0.205
Bypass-time (min)	121.3 ± 46.0	119.7 ± 35.3	0.794
Cross-clamp time (min)	54 (42.5; 69.5)	59.5 (49.8; 71.3)	0.028
On-pump CABG	140 (99.3)	78 (98.7)	1.000
Off-pump CABG	1 (0.7)	0 (0.0)	1.000
antegrade cardioplegia	54 (38.3)	32 (40.5)	0.774
antegrade + retrograde cardioplegia	85 (60.3)	45 (56.9)	0.669
Number of distal anastomoses	3 (3; 4)	3 (3; 4)	1.000
Arterial graft	84 (59.6)	53 (67.1)	0.331
LITA	82 (58.2)	52 (65.8)	0.313
Complete revascularization	116 (82.3)	58 (73.4)	0.116

STEMI, ST-elevation myocardial infarction; NSTEMI, non-STEMI; CABG, coronary artery bypass grafting; LITA, left internal thoracic artery. Values are expressed as n (%), mean ± SD or median (range).

### 3.4 Postoperative Details

As shown in Table 4, persistent low-cardiac output occurred in 20 (14.2%) STEMI and 7 (8.9%) NSTEMI patients (*p* = 0.289). An ECLS was implanted in 14 (9.9%) STEMI patients and in one (1.3%) NSTEMI patient. Most patients of either group remained mechanically ventilated for more than two days without differences between the groups (*p* = 1.000). A re-thoracotomy due to bleeding was necessary in 12 (8.5%) STEMI and 4 (5.1%) NSTEMI patients (*p* = 0.425). Neurological injuries were evident in 17 (12.1%) STEMI and 8 (10.1%) NSTEMI patients post-operatively (*p* = 0.825). Of those, strokes were present in 9 (6.4%) STEMI and 4 (5.1%) NSTEMI patients (*p* = 0.774) while 16 (11.3%) STEMI and 4 (5.1%) NSTEMI patients showed signs of hypoxic brain damage (*p* = 0.146).

**Table 4.** **Postoperative details**.

Parameter		STEMI (n = 141)	NSTEMI (n = 79)	*p*-value
Low-cardiac output		20 (14.2)	7 (8.9)	0.289
Sepsis		33 (23.4)	12 (15.2)	0.293
Ventilation >48 h		89 (63.1)	50 (63.3)	1.000
ICU >48 h		115 (81.6)	70 (88.6)	0.185
Re-thoracotomy due to bleeding		12 (8.5)	4 (5.1)	0.425
Cerbrovascular injury		17 (12.1)	8 (10.1)	0.825
	Stroke	9 (6.4)	4 (5.1)	0.774
	Hypoxic brain damage	16 (11.3)	4 (5.1)	0.146
Renal replacement therapy		56 (39.7)	22 (27.8)	0.080
>3 PRBC units		84 (59.6)	46 (58.2)	0.886
>1 TC unit		49 (34.6)	31 (39.2)	0.559
Re-myocardial infarction		3 (2.1)	0 (0)	0.554
Peak CK-MB (U/L)		247.2 (116.3; 427.8)	130.4 (63.6; 275.6)	0.001
Ventricular arrhythmias		24 (17.0)	8 (10.1)	0.231
ECLS		14 (9.9)	1 (1.3)	0.001
IABP		2 (1.4)	0 (0.0)	0.537

ICU, Intensive care unit; PRBC, packed red blood cells; TC, thrombocyte; ECLS, extracorporeal life support; IABP, intra-aortic balloon pump. Values are expressed as mean ± SD, median (range) or n (%).

### 3.5 Outcome

**Fig. 2.**
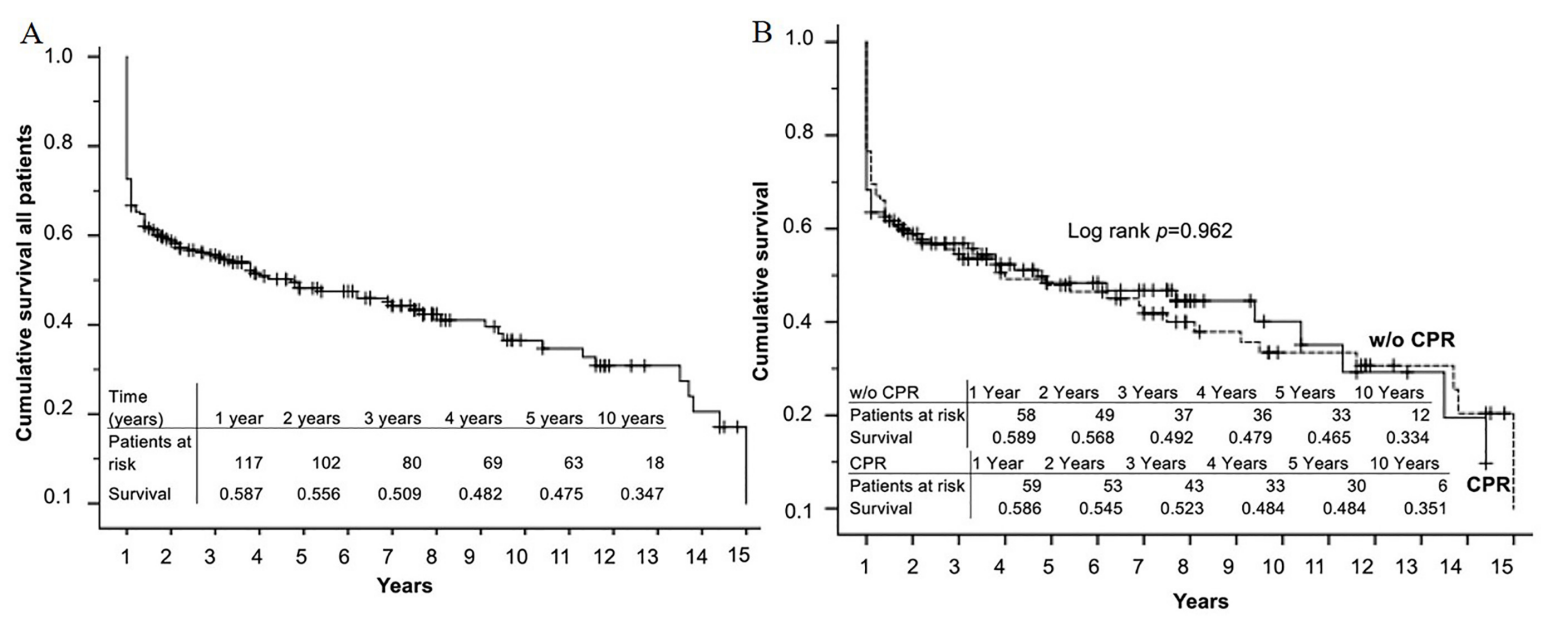
**Survival of patients with cardiogenic shock**. (A) Overall survival of all patients with cardiogenic shock. 10-year Kaplan-Meier survival curves after acute myocardial infarction and coronary artery bypass grafting in patients with cardiogenic shock. (B) Survival after cardiopulmonary resuscitation. 10-year survival of patients with pre-operative cardiopulmonary resuscitation (CPR) or without (w/o) CPR. Kaplan-Meier survival curves showed no significant differences between the groups (*p* = 0.962).

**Fig. 3.**
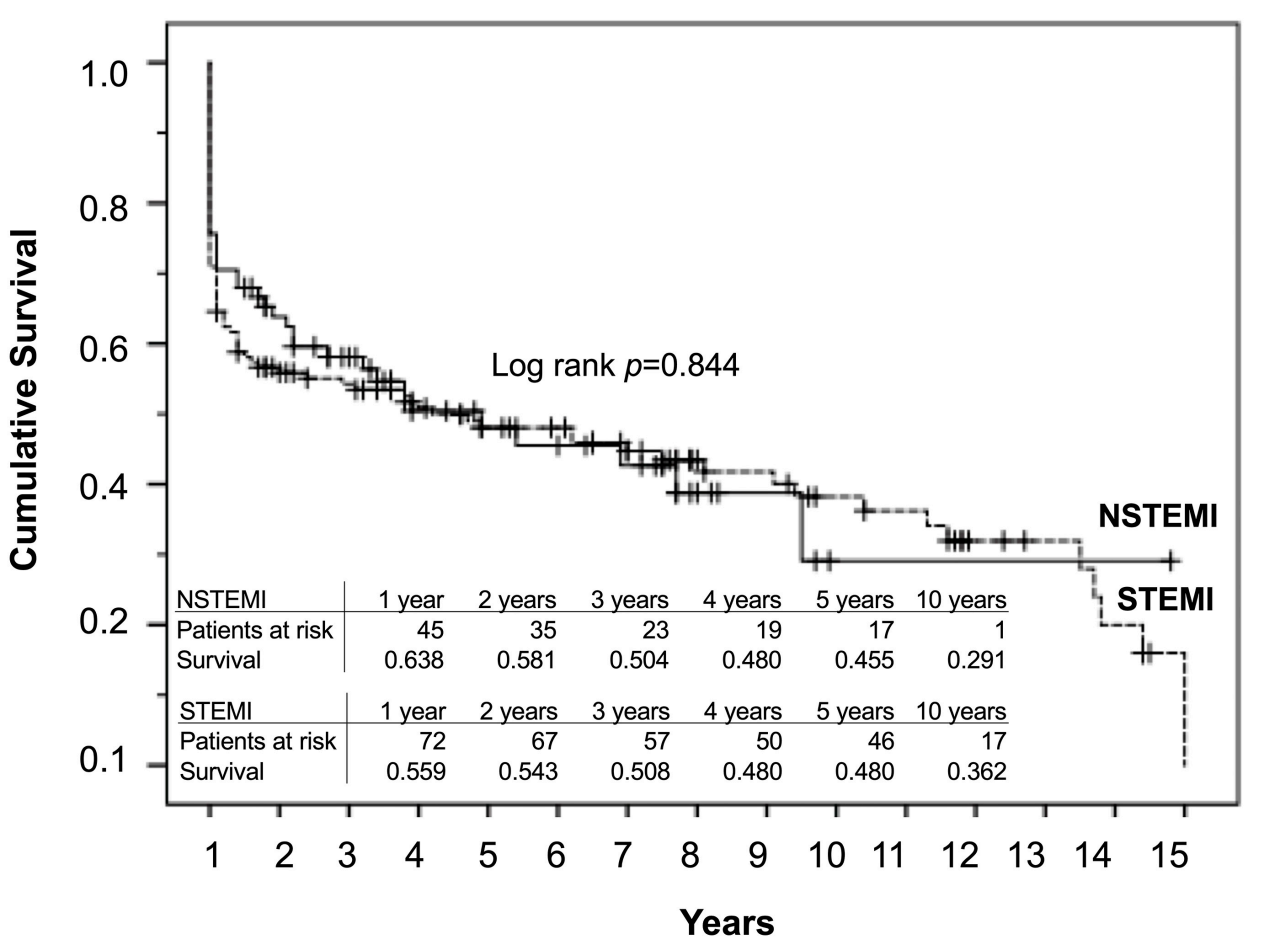
**10-year survival of STEMI and NSTEMI patients with cardiogenic shock**. Kaplan-Meier survival curves for STEMI and NSTEMI patients showed no differences between the groups (*p* = 0.844).

30-day mortality for STEMI patients was 32.6% and 31.6% for NSTEMI patients (*p* = 0.28). If patients with pre-operative cardiac arrest were excluded, 30-day mortality remained 14.2% for STEMI and 11.4% for NSTEMI cases (*p* = 0.679; Table 5). Long-term mortality covered a 10-year time period. As displayed in Fig. 2A, overall survival was 58.7% after 1 year, 47.5% after 5 years and 34.7% after 10 years. We did not find significant differences between pre-operatively resuscitated patients and those who did not suffer from cardiac arrest prior to surgery (log-rank *p* = 0.962, Fig. 2B). We also did not observe differences regarding the survival rate of STEMI versus NSTEMI patients (log-rank *p* = 0.844; Fig. 3).

**Table 5.** **Outcome**.

Parameter, n (%)	STEMI (n = 141)	NSTEMI (n = 79)	*p*-value
30-day mortality all patients	46 (32.6)	20 (31.6)	0.285
30-day mortality excluding CPR	20 (14.2)	9 (11.4)	0.679

CPR, cardiopulmonary resuscitation; Values are expressed as n (%).

### 3.6 Predictors of 30-Day Mortality

Independent risk factors for 30-day mortality included age >75 years (OR 12.603, 95% CI 3.818–41.608, *p* = 0.000) and lactate levels >8 mmol/L (OR 4.115, 95% CI 1.446–11.708; *p* = 0.008) pre-operatively, while complete revascularization positively influenced 30-day mortality (OR 0.204; 95% CI 0.058–0.712; *p* = 0.013, Table 6).

**Table 6.** **Predictors of 30-day mortality**.

Parameter	OR	95% CI	*p*-value
Age >75 years	12.603	3.818–41.608	0.000
Lactate >8 mmol/L	4.115	1.446–11.708	0.008
Complete revascularization	0.204	0.058–0.712	0.013

OR, Odds ratio; CI, confidence interval.

## 4. Discussion

Since the publication of the Should We Emergently Revascularize Occluded Coronaries for Cardiogenic Shock (SHOCK) trial by Hochman and co-workers in 1999, no further attempts have been made to clarify the role of CABG in this patient population in a prospective, randomized study [7, 11]. Although that work demonstrated that an operative revascularization achieves a better outcome compared to a conservative strategy, it is still generally assumed, that the peri-operative risk for complications would almost always exceed any possible benefit. This belief was supported by the continuous progress made regarding interventional revascularization procedures and the use of devices such as Impella® or ECLS [2, 3]. In fact, these advances led to the assumption that since the 1990s, CS patient outcome must have improved significantly and thus, a surgical approach would not be worth considering anymore. However, the results of the Culprit Lesion Only PCI versus Multivessel PCI in Cardiogenic Shock (CULPRIT-SHOCK) trial contradicted these assumptions [1]. Instead, this study underlined the persistent high rate of complications and continuing large proportion of low-output failures among CS patients with AMI, especially when complete revascularization was aimed at in patients with complex coronary artery disease (CAD) by using multi-vessel PCI. A surgical treatment group was deliberately not part of the CULPRIT-SHOCK study design, claiming that the original SHOCK trial had already demonstrated that CABG and PCI produced comparable results in CS patients. This conclusion however only holds true for patients with simple CAD [11]. In addition, only 15% of patients in the CABG group received an internal mammary bypass at the time the SHOCK trial was conducted. Given the prognostic importance of this graft, it is worth speculating that CABG in CS patients would likely produce better outcomes today than in the 1990s.

### 4.1 Impact of Revascularization Mode and Use of CPR in CS Patients on the Outcome After Emergency Revasculariation

Overall, 30-day mortality in the CULPRIT-SHOCK trial was around 50%. For comparison, contemporary surgical data—like the work presented here—repeatedly demonstrated 30-day mortality rates of around 30% [8, 9]. Of course, any comparison is limited by the fact that all surgical data are gathered retrospectively and thus, are biased. In particular, differences in CS definition or recollection of data regarding information as sensitive as neurological injury or CPR timing/length may result in profound survival differences. Nevertheless, two questions arise given the differences in survival between data on CABG in CS patients and those who were treated by PCI: first, are AMI patients with CS referred to CABG healthier than those undergoing PCI? And, second, are the actual risks associated with CABG lower than expected? Regarding question one, AMI patients with CS that cannot be revascularized by PCI are considered a high-risk population per se, with a mortality that may be higher than 70% [12]. Another major variable defining a high-risk CS patient is the need for cardiopulmonary resuscitation (CPR) prior to treatment. In the CULPRIT-SHOCK trial, 53% of all patients suffered from CA before randomization. Although our registry included only a slightly lower percentage of this particular group, 30-day mortality was markedly lower compared to the study by Thiele and co-workers. The work of Davierwala *et al*. [8] contained a stable pre-operative resuscitation rate among CS patients of around 30% over a period of 14 years. Nevertheless, in-hospital mortality was found to be reduced over time and reached 24% between 2010 and 2014. Recent data from the Society of Thoracic Surgeons (STS) Adult Cardiac Surgery Database (ACSD) analyzed the in-hospital mortality of different patient sub-groups with AMI and CS that underwent CABG within seven days after the initial event. 422 of 5496 patients were referred for immediate revascularization. While 97% of this group had undergone CPR prior to surgery, 30-day mortality was below 60% [9]. The authors of the CULPRIT-SHOCK trial discuss that the higher rate of 3-vessel disease—63% in both study arms—may have contributed to the increased rate of deaths, as these cases are knowingly associated with a worse prognosis. In contrast to these assumptions, surgical complete revascularization was independently associated with an improved 30-day survival in the study presented here. These results are in line with the original SHOCK-trial, which already indicated that CS patients with more complex CAD that were treated with CABG showed a significantly lower mortality compared to those that underwent PCI [11]. Remarkably, these results were obtained in the non-ECLS era and despite the fact that CABG patients not only suffered from more extended CAD but also had to endure a prolonged time of myocardial ischemia and potential hemodynamic instability while transported to the operating theatre.

### 4.2 No Difference between STEMI and NSTEMI after Emergency CABG

Some studies also implied that the type of myocardial infarction—STEMI or NSTEMI may have an impact on the survival of CS patients [8]. As mentioned in the current European Society of Cardiology (ESC) guidelines, data on STEMI patients with CS undergoing emergency CABG are extremely rare. The reason for that scarcity, however is a lack of data, not a large pool of evidence that STEMI patients with CS do not benefit from a surgical approach [5]. Our analysis did not reveal differences between these groups. Neither for post-operative complications nor for short- or long-term survival. However, survival alone does not define patients’ benefit from a procedure as invasive as CABG. Instead, thromboembolic events may also profoundly limit the prognosis or at least quality of life of these patients.

### 4.3 Impact of Cerebral Injury on the Outcome after Emergency CABG

Stroke rates are generally increased in patients with AMI and are higher in patients that undergo CABG than those treated with PCI. In this regard, we observed strokes in 5.2% of all patients. As the pathogenesis of cerebrovascular events is still unclear in many patients, reducing this danger remains difficult. However, the routine use of continuous near-infrared spectroscopy (NIRS) may be a tool to monitor and adjust cerebral oxygen supply intra-operatively [13]. In addition, implementation of an individualized patient blood management by point-of-care diagnostics such as Rotem® or Multiplate® may avoid inadequate transfusion that may increase the risk of thromb-embolic cerebrovascular events associated with cardiac surgery [14, 15, 16]. In the setting of CS, though, the risk of a major cerebrovascular injury is also critically influenced by the incidence of hypoxic brain damage. In the study presented here, we observed evidence of hypoxic brain damage in 9% of all patients although 47% suffered from CA prior to surgery. Combined with strokes, the overall rate of neurological injury was around 15%. In contrast, cerebral injury in CA patients primarily treated with PCI may concern up to 50% of all patients [17, 18, 19]. One might argue that surgical patients may be pre-selected in a way that leads to an under-representation of CABG subjects with a high risk of fatal neurological injury, but only a prospective study could test this hypothesis. Nevertheless, it is tempting to speculate that rapid ECLS implantation followed by complete operative myocardial revascularization in mild, on-pump controlled hypothermia and under NIRS-controlled cerebral perfusion may provide advantages compared to interventional treatment strategies in patients with CS and complex CAD. Another concern expressed during the decision-making process of whether or not emergency CABG should be performed, relates to the probability of severe bleeding complications. In general, the use of extracorporeal circulatory systems seem to have sharpened the inter-disciplinary awareness as well as acceptance that the use of these devices goes along with a certain risk of bleeding events [1]. Nevertheless, the above mentioned point-of-care tools as well as autologous re-transfusion systems have been integrated into the peri-operative patient management at many surgical centers. Thus, the portion of uncontrolled transfusion or substitution of coagulation factors can be contained [15].

## 5. Limitations

This study analysed a retrospective data collection from a single center experienced with emergency CABG in AMI patients with CS. Therefore, it remains unclear to what extent these results are transferable to other clinics. In addition, our center does not delay surgery or uses a conservative, observatory approach in patients comparable to the patients described in this study. Thus, we cannot provide such a group for comparative outcome analyses. Given these serious limitations, only prospective, randomized, multi-center studies comparing CS patients undergoing immediate CABG with a conservative, interventional or delayed surgical approach could ultimately clarify the best mode of and time point for revascularization in these highly vulnerable patients.

## 6. Conclusions

Our work indicates that in contrast to the widespread practice of denying CS patients with AMI emergency CABG, this approach could be based on the false pretense that surgery leads to devastating results most of the time. Instead, CS patients with AMI and complex CAD could benefit from immediate operative revascularization as long as myocardial ischemia is regarded as the main reason for CS. This group of patients includes individuals with a coronary anatomy that is not amendable for PCI, if PCI has failed, if complications during PCI have occurred that impair the revascularization success of if no culprit lesion could not be identified due to complex CAD with several potential causes for myocardial ischemia. No difference between CS patients with STEMI or NSTEMI should be made in this particular setting.
